# Activation of CD11b^+^ Kupffer Cells/Macrophages as a Common Cause for Exacerbation of TNF/Fas-Ligand-Dependent Hepatitis in Hypercholesterolemic Mice

**DOI:** 10.1371/journal.pone.0049339

**Published:** 2013-01-23

**Authors:** Hiroyuki Nakashima, Yoshiko Ogawa, Satoshi Shono, Manabu Kinoshita, Masahiro Nakashima, Atsushi Sato, Masami Ikarashi, Shuhji Seki

**Affiliations:** Department of Immunology and Microbiology, National Defense Medical College, Tokorozawa, Saitama, Japan; Friedrich-Alexander-University Erlangen, Germany

## Abstract

We have reported that the mouse hepatic injury induced by either α-galactosylceramide (α-GalCer) or bacterial DNA motifs (CpG-ODN) is mediated by the TNF/NKT cell/Fas-ligand (FasL) pathway. In addition, F4/80^+^ Kupffer cells can be subclassified into CD68^+^ subset with a phagocytosing capacity and CD11b^+^ subset with a TNF-producing capacity. CD11b^+^ subset increase if mice are fed high-fat and cholesterol diet (HFCD). The present study examined how a HFCD affects the function of NKT cells and F4/80^+^ CD11b^+^ subset and these hepatitis models. After the C57BL/6 mice received a HFCD, high-cholesterol diet (HCD), high-fat diet (HFD) and control diet (CD) for four weeks, the HFCD mice increased surface CD1d and intracellular TLR-9 expression by the CD11b^+^ population compared to CD mice. Hepatic injury induced either by α-GalCer or CpG-ODN was more severe in HCD and HFCD mice compared to CD mice, which was in proportion to the serum TNF levels. In addition, liver cholesterol levels but not serum cholesterol levels nor liver triglyceride levels were involved in the aggravation of hepatitis. The FasL expression of NKT cells induced by both reagents was upregulated in HFCD mice. Furthermore, the liver mononuclear cells and purified F4/80^+^ CD11b^+^ subset from HFCD mice stimulated with either reagent *in vitro* produced a larger amount of TNF than did those from CD mice. Intracellular TNF production in F4/80^+^ CD11b^+^ cells was confirmed. The increased number of F4/80^+^ CD11b^+^ Kupffer cells/macrophages by HFCD and their enhanced TNF production thus play a pivotal role in TNF/NKT cell/FasL dependent hepatic injury.

## Introduction

The liver has a large number of macrophage lineage cells, Kupffer cells, which make up 80% of the total body macrophages [1]–[3]. Mouse liver mononuclear cells (MNCs) also have large proportion of innate immune lymphocytes, comprising NK cells (10–20%) and natural killer T (NKT) cells (15–25%) [2], [3]. These innate immune cells in the liver normally play a pivotal role in the host defense against microbes and tumors via the T helper 1 immune response [2], [4], [5]. In addition, hepatocytes produce acute phase proteins (including CRP) and complement components which are critical for the innate immune responses [2]. Indeed, synthetic CRP injection into normal and immunocompromised mice increased the phagocytic activity of Kupffer cells and protected mice from lethal bacterial infections [6].

On the other hand, liver NKT cells are also involved in liver injury. We previously reported that mouse liver NKT cells activated by α-GalCer (a synthetic glycolipid and NKT cell ligand) express Fas-ligand (FasL) and induce hepatocyte injury in a TNF/FasL-dependent manner [4], [5]. NKT cells activated by a Toll-like receptor-9 (TLR-9) agonist common bacterial DNA motifs (CpG-ODN) [7], [8] also induce hepatic injury in a TNF/FasL-dependent manner [9], which is also inhibited in NKT cell-deficient mice [9].

We have also previously demonstrated that mouse F4/80^+^ Kupffer cells can be subclassified into two major subsets according to their phenotype and function [10]. One is the CD68^+^ subset with potent reactive oxygen species (ROS) production and phagocytic capacities, and the other is the CD11b^+^ subset, with a potent capacity to produce T helper 1 cytokines (TNF, IL-12) [10]. Although CD68 protein is recognized as intracellular protein, CD68^+^ subset (not CD11b^+^ subset) indeed express surface CD68 [10]. In addition, CD68^+^ Kupffer cells may firmely adhere sinusoidal endothelial cells or hepatocytes because they were mainly present in mid-zonal zone and were hardly obtained without collagenase treatment of the liver tissues, whereas CD11b^+^ Kupffer cells/macrophages were equally present and were easily obtained without collagenase treatment. Therefore, we suggested that CD68^+^ subset may be fixed or resident Kupffer cells and CD11b^+^ Kupffer cells/macrophages (hereafter, Kupffer cells) may be migrated from the bone marrow or spleen, especially in inflammatory conditions of the liver. Klein et al. proposed the existence of two types of Kupffer cells, bone marrow derived population and non-bone marrow derived population [11]. The former population which infiltrate into liver in inflammatory response seems to be equivalent to CD11b^+^ subset, and the latter ‘sessile’ population to be equivalent to CD68^+^ subset. In addition, the injection of gadolinium chloride (GdCl_3_) or clodronate liposomes into mice depletes only CD68^+^ Kupffer cells, but not CD11b^+^ Kupffer cells [10]. Holt et al. identified two distinct subsets of F4/80^+^ hepatic macrophages in acetaminophen-induced liver injury [12]. They also demonstrated that clodronate liposome administration did not eliminate CD11b^high^F4/80^low^ subset, whereas the other CD11b^low^F4/80^high^ subset was almost completely depleted. We consider the former population corresponds to CD11b^+^ Kupffer cells and the latter population corresponds to CD68^+^ Kupffer cells.

Furthermore, we have recently demonstrated that the population of F4/80^+^CD11b^+^ Kupffer cells increases in mice fed a HFCD, and the amount of cholesterol, rather than that of triglycerides, in the diet is responsible for the increase in the number of these cells [13]. Namely, the proportions of CD11b^+^Kupffer cells in the livers of mice fed four diets were CD<HFD<HCD≤HFCD, which were also proportional to the total cholesterol levels in the liver [13]. In contrast, the proportions of NKT cells in the liver appear to gradually decrease in the same order, however, this was the result of activation-induced downregulation of NK1.1 Ag expression by NKT cells [13]. Therefore, the proportion of α-GalCer dimer-positive NKT cells was not changed by the HFCD [13]. Interestingly, the NK cells greatly increased in proportion and number in HFCD mice compared to CD mice (up to two-fold). Such activation of CD11b^+^ Kupffer cells, liver NKT cells and NK cells in the mice on the HFCD exhibited increased antitumor cytotoxicities of NK/NKT cells, but conversely, had enhanced susceptibility to endotoxin shock and the generalized Shwartzman reaction due to the increased production of TNF and IFN-γ from Kupffer cells/macrophages and NK/NKT cells, respectively [13].

In the present study, we investigated how a HFCD affects the function of CD11b^+^ Kupffer cells, NKT cells and TNF/FasL-dependent hepatitis evoked by these cells, and discuss the possible role of these cells in human non-alcoholic steatohepatitis (NASH).

## Materials and Methods

The Ethics Committee of Animal Care and Experimentation, National Defense Medical College, Japan, approved all requests for animals and the intended procedures of the present study (Permission number: 12039).

### Mice and reagents

Male C57BL/6 (B6) mice (7 weeks of age) were purchased from Japan SLC (Hamamatsu, Japan). Mice received a HFCD (15% fat, 1.25% cholesterol) (ORIENTAL YEAST co. Ltd., Tokyo), HCD (1.25% cholesterol), HFD (15% fat) or CD (normal chow) for 4 weeks before experiments. In some experiments, mice fed either HFCD or CD for 3 months were used (at around 20 weeks age).

The α-Galactosylceramide (α-GalCer) [4], [14], [15] was provided by the Pharmaceutical Research Laboratory of Kirin Brewery Company, and mice were injected intravenously (i.v.) with α-GalCer at 100 µg/kg body weight. The bacterial DNA motifs, CpG-oligonucleotides (ODN) (HC4033): TCCATGACGTTCCTGATGCT, were purchased from Hycult Biotechnology (Uden, Netherlands). Mice were i.v. injected with 10 µg/g body weight of CpG-ODN.

### Isolation of liver MNCs

Liver MNCs, including Kupffer cells, were prepared as described previously with minor modifications [4]. Briefly, liver specimens were minced thoroughly with scissors. After adding 0.5 mg/ml collagenase (WAKO, Japan) solution, specimens were shaken for 15 min in a 37°C constant-temperature bath. Next, the liver specimens were filtered through a stainless steel mesh and remnants were dissolved using rubber stick on the mesh. Thereafter, the liver MNCs were obtained by the isotonic 33% Percoll (SIGMA Chemical Co., USA) solution method. Liver MNCs without CD68^+^Kupffer cells were obtained without collagenase treatment by 33% Percoll solution.

### Measurement of serum total cholesterol (TC), high-density lipoprotein cholesterol (HDL-C), serum alanine aminotransferase (ALT), and cytokine levels

Blood samples were collected from the retro-orbital sinus at specified time points. The TC and ALT levels were measured using a DRICHEM 3000 V instrument (Fuji Medical Systems, Tokyo). ELISA kits for IFN-γ (BD PharMingen, USA) and for TNF (Endogen) were used.

### Measurement of liver cholesterol (TC) and triglyceride (TG) levels

Liver specimens were filtered through stainless steel mesh with rubber stick, and centrifuged at 430 g. The supernatant was collected and subjected to cholesterol and triglyceride analysis. Cholesterol level was measured by ultracentrifugation method and total lipoprotein level was assessed. Triglyceride was measured using a DRICHEM 3000 V instrument (Fuji Medical Systems, Tokyo).

### Flow cytometry

The MNCs were incubated for 10 min at 4°C with Fc-blocker (2.4 G2; BD PharMingen, USA) to prevent any nonspecific binding. For identification of Kupffer cells, MNCs were stained with a FITC-labeled anti-F4/80 antibody (Ab) (eBioscience, USA), PE-labeled CD11b Ab (M1/70, BD PharMingen), and biotin-labeled CD68 Ab (FA-11, SEROTEC, Oxford, UK) and developed with Cy5-streptavidin. Biotin-labeled CD1d Ab and biotin-labeled FasL Ab (eBioscience) were developed with PE and PC5, respectively. The biotin-labeled TLR-9 Ab (Hycult Biotech) was developed with PE. A flow cytometric analysis was performed using a Cytomics FC500 instrument (Coulter). For the identification of NKT cells, α-GalCer-loaded CD1-d-IgG1dimer (developed with-PE-conjugated anti-rat IgG1 Ab) (BD Pharmingen) and αβTCR Ab, and in some cases we use Vβ8 Ab and αβTCR Ab. For intracellular TNF staining, liver MNCs were isolated from the livers of mice without collagenase treatment 30 minutes after i.v. administration with α-GalCer. The cells were restimulated *in vitro* with 100 ng/ml α-GalCer for 3 h with monensin (BD Pharmingen) to block cytokine release. The cells were collected and stained for the surface expression of F4/80/CD11b or Vβ8/αβTCR. Following the permeabilization of the cell membrane, intracellular TNF were stained by PE-anti-TNF Ab (BD Pharmingen).

### In vivo cell depletion of NK cells or both NK cells and NKT cells

To deplete NK cells, anti-asialoGM1 Ab (100 µg) was injected i.p. 6 days and 3 days before the experiments. Similarly, to deplete both NK cells and NKT cells, anti-NK1.1 Ab (500 µg) (PK136) was injected i.p. 6 and 3 days before the experiments.

### Statistical analysis

The data are presented as the mean values ± SE. The statistical analyses were performed using a GraphPad Prism 5 software package (GraphPad Software, La Jolla, CA). Statistical evaluations were compared using the standard one-way analysis of variance followed by the Bonferroni post-hoc test. *P*<0.05 was considered to indicate a significant difference.

## Results

### Body weight, serum and liver cholesterol (TC) and triglyceride (TG) levels

As we recently reported [13], the average body weight of mice that received a HFCD or HFD for 4 weeks (from 7 to 11 weeks of age) was significantly higher than the weight of CD mice and HCD mice (Fig. S1a). The TG levels of liver homogenates from the HFCD and HFD mice, but not of HCD mice, were higher than those of the CD mice (Fig. S1b). Serum TG levels showed a same tendency (not shown). The serum TC levels of HFCD mice and HFD mice were higher than those of CD and HCD mice (Fig. S1c). The liver TC levels of HFCD mice and HCD mice were higher than those of CD mice and HFD mice (Fig. S1d).

### Hepatocyte injury and serum TNF levels induced by either α-GalCer or CpG-ODN are markedly increased in HCD and HFCD mice

**Figure 1:**
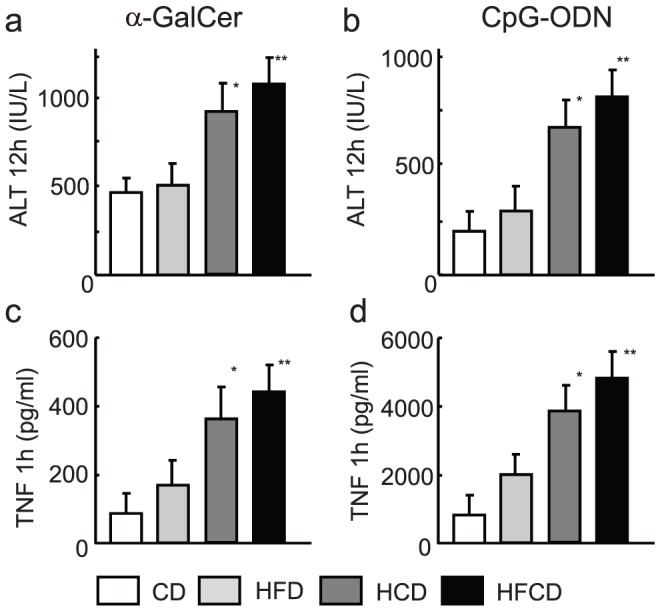
The serum ALT and TNF levels in mice after α-GalCer or CpG-ODN injection. HFCD and CD mice were injected i.v. with either α-GalCer or CpG-ODN, and the serum levels of ALT (12 h after reagent injection) (**a, b**) and TNF (1 h after reagent injection) (**c, d**) were examined. The data shown are the means ± SE from eight mice in each group. *P<0.05 vs. CD and HFD, ** *P*<0.01 vs. CD and HFCD.

The serum ALT levels at 12 h after α-GalCer or CpG injection and TNF levels at 1 h after α-GalCer or CpG injection into HCD or HFCD mice were significantly higher than those in CD and HFD mice (Figs. 1a–d). The TNF levels at all other time points were very low or undetectable.

### Serum IL-12, IL-10 and IL-6 levels after either α-GalCer or CpG-ODN injection

**Figure 2:**
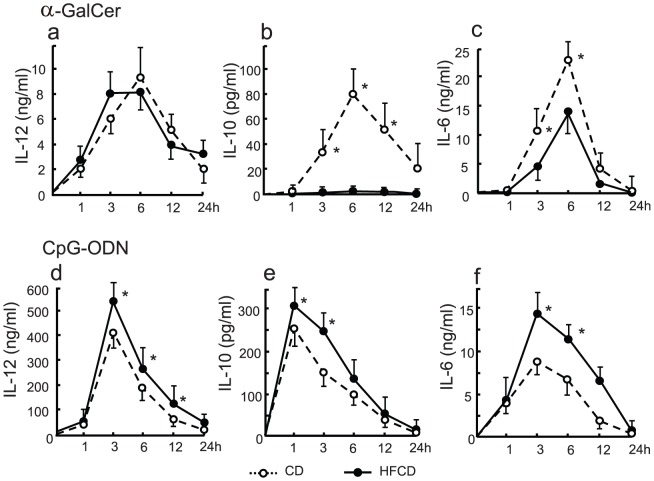
Serum IL-12, IL-10 and IL-6 levels after either α-GalCer or CpG-ODN injection. The HFCD and CD mice were injected i.v. with either α-GalCer or CpG-ODN, and the serum levels of cytokines at the indicated times were examined. The data shown are the means ± SE from three mice in each group.*P<0.01 vs. CD and HFCD.

The serum IL-12 levels increased after α-GalCer injection, but the levels did not differ at the indicated time points between CD mice and HFCD mice (Fig. 2a). IL-10 and IL-6 levels of HFCD mice were significantly lower than those in CD mice, especially IL-10 levels did not elevate in HFCD mice (Fig. 2b, c). The serum IL-12, IL-10 and IL-6 levels of HFCD mice after CpG injection at indicated time points were higher than those of CD mice (Fig. 2d–f).

### The histological findings of experimental hepatitis

**Figure 3:**
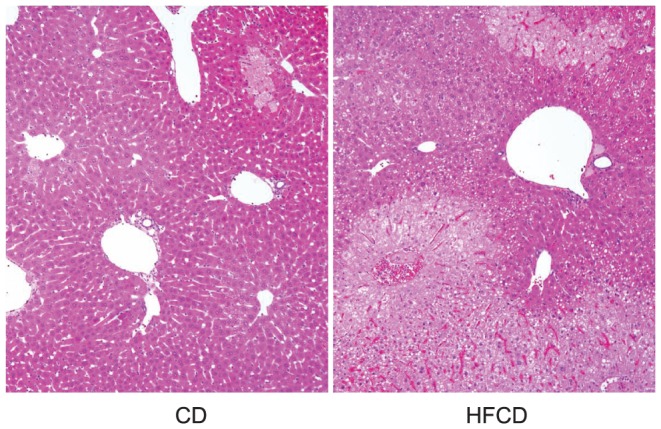
Histological findings of α-GalCer hepatic injuries. The livers from a CD and a HFCD mouse 24 h after α-GalCer injection (100×). Livers were fixed in 10% formalin for hematoxylin-eosin staining. Data are the representative findings from five mice with similar results.

**Figure 4:**
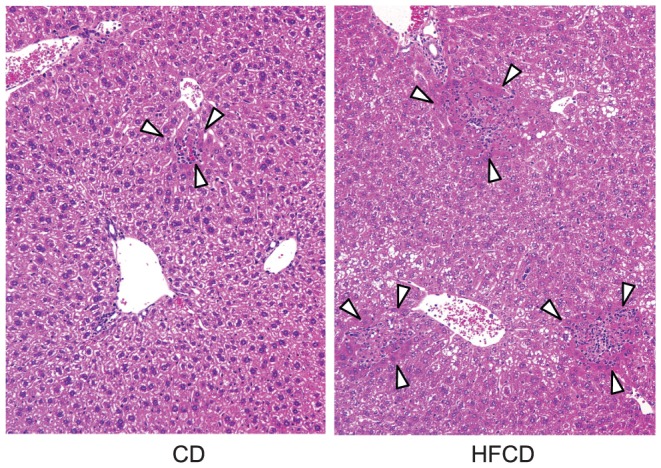
Histological findings of CpG hepatic injuries. The livers from CD mice and HFCD mice 24 h after CpG injection (200×). The livers were fixed in 10% formalin for hematoxylin-eosin staining. Data are the representative findings from five mice with similar results.

Our recent study demonstrated that microvesicular steatosis was found in the hepatocytes of HFCD mice, which was not found in the CD mice and was less obvious in HFD mice and HCD mice [13]. The liver of HFCD mice after α-GalCer injection showed large degenerative areas as compared to those of CD mouse (Fig. 3). In the case of CpG injection, the liver of the HFCD mice showed more degenerative (necrotic) areas with leukocyte infiltration than those in the CD mouse (Fig. 4, indicated by arrow heads). Similar results were obtained in several other mice after α-GalCer or CpG injection.

### Proportional and phenotypic changes of F4/80^+^Kupffer cells cells in the liver MNCs

**Figure 5:**
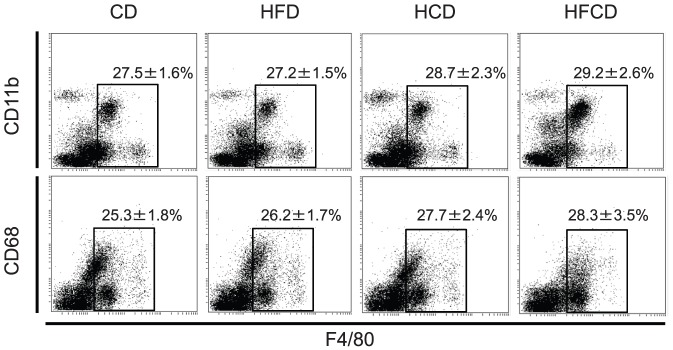
F4/80 and either CD11b or CD68 expression of whole liver MNCs. Liver MNCs were obtained from CD, HFD, HCD and HFCD mice and expression of F4/80 either with CD11b or CD68 were examined. The numbers were the means±SE from four mice in each group. F4/80 positive gate was determined by using the isotype control Ab.

**Figure 6:**
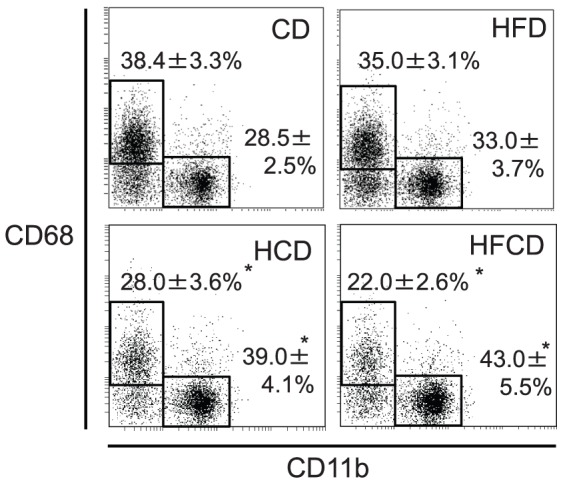
The expression of CD68 and CD11b by liver F4/80^+^ Kupffer cells. Liver MNCs were obtained from CD, HFD, HCD and HFCD mice, and Kupffer cells gated by F4/80 were analyzed for their expression levels of CD68 and CD11b. CD68 positive gate was determined by using the isotype control Ab. The numbers are the means±SE from six to eight mice in each group. *P<0.05 vs. CD and HFD.

**Figure 7:**
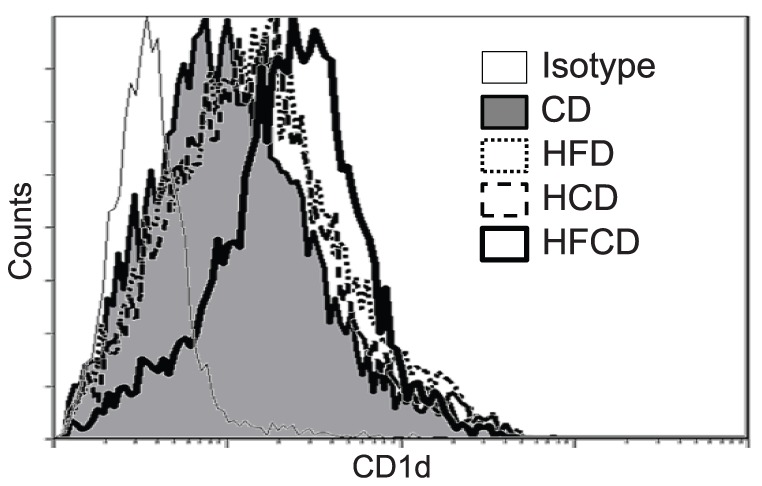
CD1d expression by CD11b Kupffer cells/macrophages. Liver MNCs were obtained from CD, HFD, HCD and HFCD mice, and Kupffer cells gated by F4/80 and CD11b were analyzed for their expression of CD1d. The data shown are representatives of five mice with similar results.

**Figure 8:**
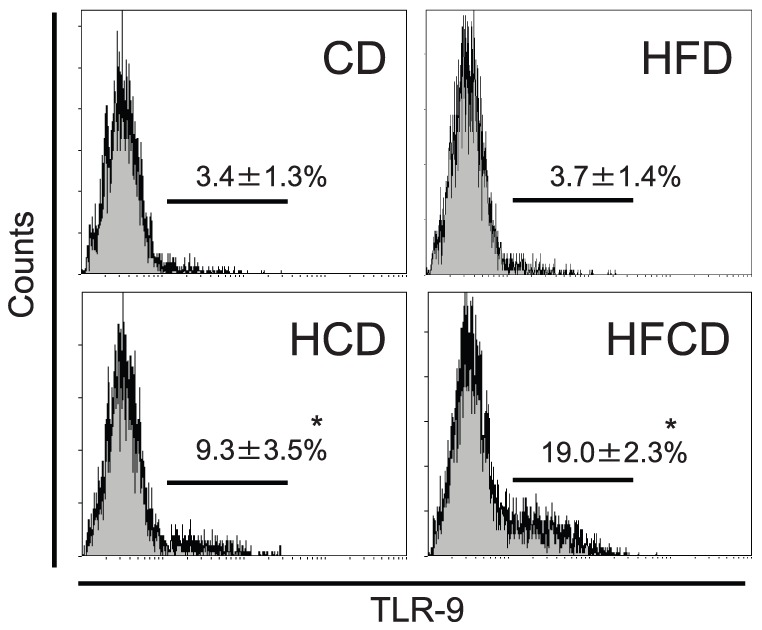
TLR-9 expression of CD11b Kupffer cells/macrophages. Liver MNCs were obtained from CD, HFD, HCD and HFCD mice, and Kupffer cells gated by F4/80 and CD11b were analyzed for their expression of TLR-9. The data are the means±SE from five mice in each group. *P<0.05 vs. CD and HFD.

Both F4/80 and CD11b staining and F4/80 and CD68 staining showed that F4/80^+^ cells tend to increase in HFCD mice, but the difference is not significant (Fig. 5). F4/80 positive gate was determined carefully by the isotype control Ab. However, as we recently reported [13], the number/proportion of F4/80^+^CD68^−^CD11b^+^ Kupffer cells increased, and the number/proportion of F4/80^+^CD68^+^CD11b^−^ Kupffer cells decreased in HFCD and HCD mice relative to those cells in CD mice and HFD mice (Fig. 6). In addition, the surface CD1d expression (Fig. 7) and intracellular expression of TLR-9 increased in the F4/80^+^ CD11b^+^ Kupffer cells from HCD and HFCD mice (Fig. 8). However, the CD1d expression of F4/80^+^CD68^+^ cells may slightly decrease in HFCD mice, although they express high levels of CD1d as compared to F4/80^+^CD11b^+^ cells (Fig.S2). The proportion of CD11c^+^ cells in CD68 Kupffer cells in HFCD mice was larger than that in CD mice (35. 4±2.2% vs 25.2±1.4%, n = 3, p<0.05) and the proportion in CD11b Kupffer cells/macrophages in HFCD mice was also larger than that in CD mice (26.6±2.7% vs 18.4±1.6%, n = 3, p<0.05), and the CD11c intensities (MFI) tended to increase in both subsets.

### *In vitro* TNF production from liver MNCs and F4/80^+^CD11b^+^Kupffer cells stimulated with α-GalCer or CpG

**Figure 9:**
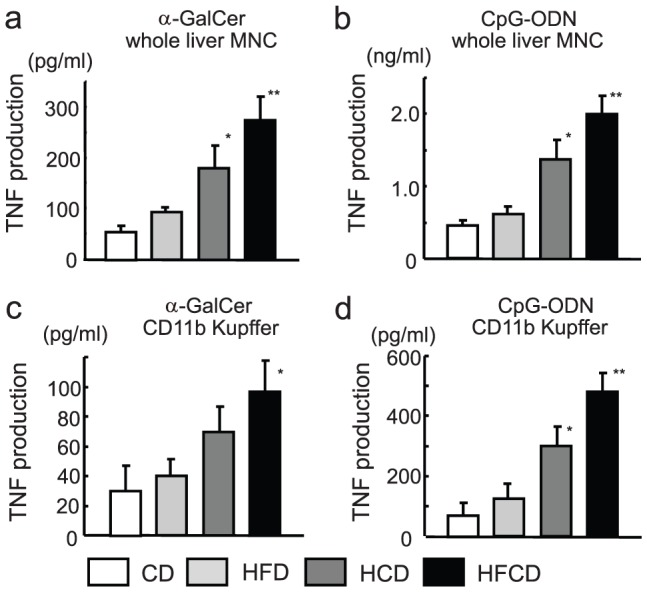
*In vitro* TNF production by liver MNCs or CD11b^+^ Kupffer cells/macrophages from CD and HFCD mice. (**a, b**) Liver MNCs were obtained from the four groups of mice, and 5×10^5^ MNCs/200 µl were stimulated with either α-GalCer (100 ng/ml) or CpG (20 µg/ml) in a 96 well plate for 6 h. Supernatants were examined for TNF. The data shown are the means ± SE from five mice, *P<0.05 vs. CD and HFD, ** *P*<0.01 vs. CD and HFD. (**c, d**) Liver MNCs were obtained from eight to ten mice without collagenase digestion and F4/80^+^ cells (mostly CD11b^+^ Kupffer cells/macrophages) were purified by a MACS sorting system. A total of 5×10^5^ CD11b^+^ population/200 µl were stimulated with either α-GalCer or CpG in a 96 well plate for 6 h. The supernatants were examined for TNF. The data shown are the means ± SE from four independent experiments. *P<0.05 vs. CD and HFD, ** *P*<0.01 vs. CD and HFD.

To determine whether the liver MNCs themselves are activated by HFCD, the cells from all mouse groups were stimulated with either α-GalCer or CpG. The liver MNCs from HFCD mice and HCD mice stimulated with each stimulus produced a much larger amount of TNF than did the liver MNCs from CD mice and HFD mice (Figs. 9a, b).

We also examined the TNF production of sorted F4/80^+^ CD11b^+^ Kupffer cells in HFCD mice and CD mice. When Liver MNCs were collected without collagenase treatment, F4/80^+^ cells were mostly CD11b^+^ but not CD68^+^ [10]. Such F4/80^+^Kupffer cells were sorted by magnetic beads (approximately 90% were F4/80^+^CD11b^+^Kupffer cells) and were stimulated with reagents *in vitro*. The CD11b^+^ Kupffer cells from HFCD mice stimulated by either reagent produced a greater amount of TNF after stimulation than did the cells isolated from CD mice and HFD mice (Figs. 9c, d), which may have been due to the enhanced expression of CD1d and TLR-9 by CD11b^+^ Kupffer cells (Figs. 7, 8). Although the sorted CD11b^+^ Kupffer cells stimulated with each of the reagents alone produced smaller amounts of TNF than did the whole liver MNCs, both the magnetic sorting procedure and the absence of liver lymphocytes and their products (mainly IFN-γ) may impair Kupffer cell function. Nevertheless, these results suggest that HFCD mice not only have proportionally increased CD11b^+^Kupffer cells, but also augment their TNF-producing capacity.

To further substantiate the TNF production from CD11b^+^Kupffer cells, liver MNCs from CD mice depleted of NKT cells and/or NK cells were stimulated with α-GalCer *in vitro*. TNF production was not affected by depletion of both NK cells and NKT cells (by anti-NK1.1 Ab injection) or depletion of NK cells alone (by anti-asialoGM1 Ab) (Fig. S3a). However, IFN-γ and IL-4 production was significantly decreased by depletion of both NK cells and NKT cells, but not by the depletion of NK cells (Fig. S3b, c). IFN-γ and IL-4 are known to be cytokines produced by α-GalCer-activated NKT cells [4], [5]

In addition, sorted liver MNCs from HFCD mice showed that F4/80^+^CD11b^+^ Kupffer cells produce a large amount of TNF after α-GalCer stimulation (at 6 h) but F4/80^−^ CD11b^−^ lymphocytes did not produce TNF (if any) (Fig. S3d). However, F4/80^−^ CD11b^−^ lymphocytes produced a large amount of IFN-γ and IL-4 after α-GalCer stimulation (at 24 h) presumably because NKT cells stimulated with α-GalCer presented by CD1d-expressing B cells (Fig. S3e, f). Somewhat unexpectedly, the CD11b^+^ Kupffer cells produced a substantial amount of IFN-γ (Fig. S3e).

### Production of intracellular TNF by CD11b^+^ Kupffer cells

**Figure 10:**
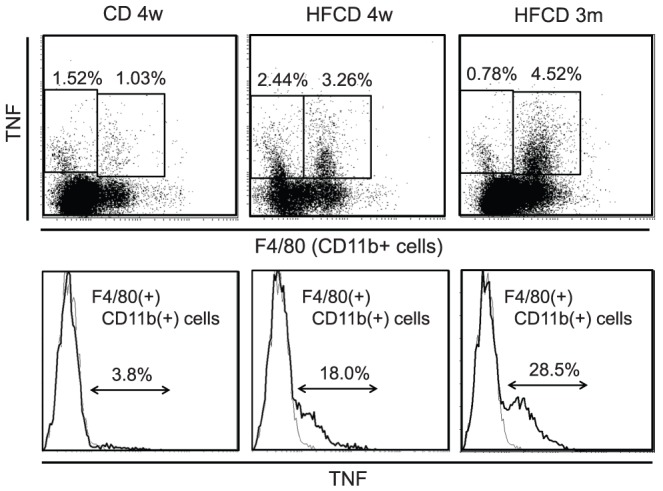
Production of intracellular TNF by liver MNCs. Liver MNCs from CD mice, HFCD mice and HFCD mice (3 months) were obtained 30 min after α-GalCer injection without collagenase treatment and liver F4/80 (CD11b)^+^ cells were examined for their intracellular TNF expression as described in materials and methods. Data are the representative findings from three to five mice with similar results.

In addition to CD mice and HFCD mice, mice fed HFCD for 3 months were used, because we previously reported that serum TNF levels after α-GalCer injection increased in an age dependent manner [16]. 30 min after α-GalCer injection, liver MNCs were obtained without collagenase treatment. 3.8% in CD11b^+^ cells in CD mice expressed intracellular TNF (Fig. 10), 18.0% in F4/80/CD11b^+^ cells in HFCD mice expressed intracellular TNF (Fig. 10) and 28.5% of F4/80/CD11b^+^ cells in HFCD mice (3 months) expressed intracellular TNF (Fig. 10). Becaus of the technical difficulty, for detection of intracellular TNF of NKT cells, Vβ8TCR Ab was used (approximately 70% of NKT cells are Vβ8^+^) [17]. NKT cells (Vβ8^+^ cells with intermediate TCR) may produce TNF, but the proportions of TNF-producing NKT cells were relatively low regardless of mouse groups (Fig. S4). T cells, NK cells and B cells did not show intracellular TNF (not shown). These results suggest that F4/80/CD11b^+^ Kupffer cells/macrophages are main TNF producers after α-GalCer injection, especially in HFCD mice.

### The activation of liver NKT cells in HFCD mice

**Figure 11:**
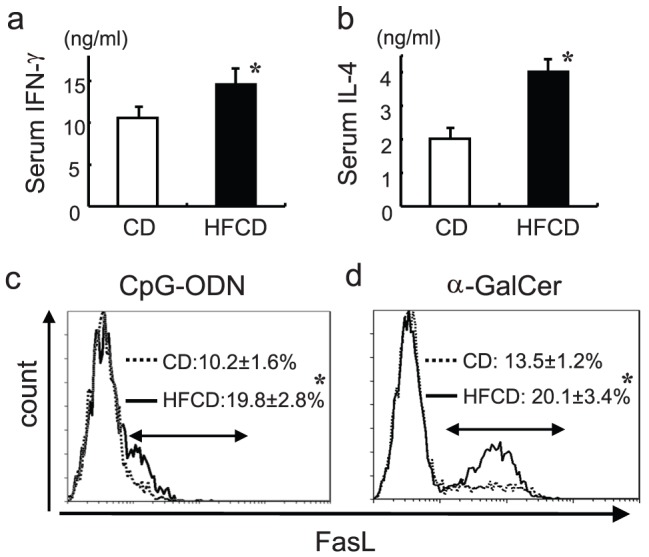
The serum IFN-γ and IL-4 levels after α-GalCer or CpG injection in mice, and the FasL expression of NKT cells. HFCD mice and CD mice were injected i.v. with α-GalCer and the serum levels of cytokines at 6 h after injection were measured (**a, b**). The data are the means±SE from five mice in each group. Mice were injected with CpG, and the FasL expression of NKT cells was examined at 1 h after injection (**c**). In other experiments, mice were injected i.p. with IL-12 (24 h before) and were injected i.v. with α-GalCer, and the FasL expression of NKT cells was examined 1 h after α-GalCer injection (**d**). The percentages are the means ± SE from four mice in each group. *P<0.05 vs.CD.

The serum IFN-γ levels, as well as the IL-4 levels, at 6 h after α-GalCer injection were significantly higher in the HFCD mice than those in CD mice (Fig. 11a, b), both of which are known to be cytokines produced by ligand-activated NKT cells and an indicator of NKT cell activation [4], [5]. Since the differences in the FasL expression levels of liver NKT cells increased significantly after α-GalCer injection in the HFCD mice, but the differences were generally less pronounced between HFCD and CD mice, the mice were pretreated with interleukin-12 (IL-12) (24 h before) and injected with α-GalCer to augment the FasL expression of the NKT cells, as we have previously reported [17]. Consequently, the IL-12 pretreatment markedly aggravated hepatitis, as shown by the increased ALT levels after α-GalCer injection in HFCD mice compared to control mice (7, 500 v.s 1, 600), and the FasL expression on NKT cells was obviously increased in the HFCD mice compared to that of CD mice (Fig. 11d). The FasL expression on NKT cells 1h after CpG injection was also significantly increased in the HFCD mice compared to that of CD mice (Fig. 11c). Because NKT cells started to disappear by downregulation of their TCR and NK1.1 around 1 h after α-GalCer injection, liver MNCs were gated by α-GalCer-loaded CD1-d-IgG1dimer and αβTCR Ab and were analyzed for their FasL expression at this time point (1 h after injection). These results confirm that the FasL levels on NKT cells correlate with the severity of hepatic injury induced by α-GalCer or CpG.

### The effects of HFCD/CD on the liver injury

**Figure 12:**
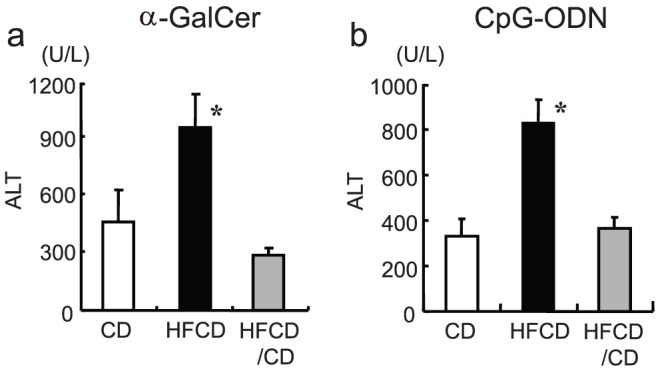
Improvement in the hepatic injury by HFCD/CD. The mice received HFCD for four weeks and then the HFCD diet was changed to a CD diet (HFCD/CD). After four weeks, the mice were injected with either α-GalCer or CpG-ODN, and the serum ALT levels were examined. The data shown are the means ± SE from six to ten mice in each group. *P<0.05 vs. other groups.

The mice received HFCD for four weeks, and then the HFCD diet was changed to a CD diet (HFCD/CD). After four weeks, the serum TC levels and body weight were reversed to levels similar to those in CD mice (not shown). The ALT levels in the HFCD/CD mice after either α-GalCer or CpG injection also were reversed to levels similar to those in CD mice (Fig. 12a, b). The proportion of CD11b^+^ subset in the total F4/80 Kupffer cell population in the livers of HFCD/CD mice also reverted to the normal proportion (Fig. S5).

## Discussion

It is known that α-GalCer is a synthetic ligand of NKT cells that was originally identified in a marine sponge [14], [15], and specifically activates NKT cells in the presence of macrophages/dendritic cells. Macrophages/Kupffer cells which endocytose α-GalCer not only activate themselves to produce IL-12 and TNF, but also present α-GalCer to the Vα14Jα18/Vβ8 T cell receptors of NKT cells with their CD1d molecules [18], [19]. The liver NKT cells activated with α-GalCer produce IFN-γ (as well as IL-4) to activate NK cells and CD8^+^ CD122^+^ T cells to acquire antitumor cytotoxicity, and tumor-specific CD8^+^ T cells are finally induced [4], [20]. On the other hand, NKT cells activated by the TNF produced by Kupffer cells express FasL and induce hepatocyte injury in a TNF/FasL/Fas-dependent manner [4].

Unmethylated CpG-ODN motifs (GACGTT for mice and GTCGTT for humans) are common bacterial DNA and are agonists of TLR-9 in macrophages and Kupffer cells; bacteria and their DNA fragments can stimulate innate immune responses through macrophages/Kupffer cells [7], [21]. Therefore, every gram positive and negative bacterium has the potential to stimulate liver innate immune cells through Kupffer cells. CpG-ODN injection into mice activates Kupffer cells to produce IL-12 and TNF. The former activates NK cells to acquire antitumor activity, and the latter activates NKT cells to express FasL [9]. Therefore, hepatic NK cells are the main antitumor effectors, but hepatic NKT cells expressing FasL become hepatotoxic effectors after CpG-ODN injection [9]. We noted that the hepatic injury was largely inhibited in Fas-L deficient *gld/gld* mice, NKT cell-deficient CD1d-/- mice and TNF-depleted mice [9]. Thus, in contrast to α-GalCer, CpG-ODN is not a specific activator of NKT cells, but FasL-expressing NKT cells activated by TNF are common effectors for hepatic injury induced by either reagent.

In the present study, we showed that the liver cholesterol levels, but not the serum cholesterol levels nor liver triglyceride levels, are related to the degree of hepatocyte injury induced by either α-GalCer or CpG-ODN. The body weight gain induced by HFCD or HFD is also not directly related to the severity of the hepatitis. Furthermore, increased liver cholesterol accumulation by HFCD or HCD not only increased the number of CD11b^+^ Kupffer cells in the population of liver MNCs but also augmented the TNF production of CD11b^+^ cells stimulated by either of the reagents. The HFCD elevated the serum IFN-γ and IL-4 levels and upregulated the FasL expression of liver NKT cells after α-GalCer injection more than was observed in CD mice. The increased CD1d and TLR-9 expression by the CD11b CD11b+ Kupffer cells by the HFCD may explain these phenomena.

We have recently reported that the HFCD activates liver innate immune MNCs including Kupffer cells, NK cells, NKT cells and CD8^+^ CD122^+^ T cells [13]. The CD69 activation Ag was upregulated in these liver lymphocytes, and the NK1.1 expression of NKT cells was downregulated due to their activation [13]. The liver CD11b^+^ Kupffer cells, NK cells and CD8^+^ CD122^+^ T cells increased in number and proportion [13]. Such changes evoked opposite immunologic outcomes in mice. On one hand, the HFCD activated the liver MNCs (partly due to increased basal serum IL-12 levels) and augmented the antitumor cytotoxicity and antitumor immunity of liver NK/NKT cells with/without exogenous IL-12 or LPS injection because of their increased IFN-γ production, resulting in the resistance of mice to liver tumor metastasis. On the other hand, the activation also conversely renders mice susceptible to LPS-induced shock and the generalized Shwartzman reaction, which was suggested to be related to the increase in CD11b^+^ Kupffer cells and their TNF production [13]. The NK/NKT cells in HFCD mice may also be involved in the aggravated multi-organ dysfunction in LPS-induced shock as a result of their perforin-dependent cytotoxicity against vascular endothelial cells [13]. Furthermore, the present study revealed that HFCD upregulates the CD1d and TLR-9 expression of CD11b^+^Kupffer cells and the ligand-stimulated TNF production of these cells, which subsequently increases the FasL expression of NKT cells, and thereby aggravates hepatic injuries. In chronic carbon tetrachloride administration, bone marrow derived CD11b^+^F4/80^+^ macrophage population infiltrates to liver and reportedly contributes to progression of hepatic fibrosis [22]. This population seems to be identical to CD11b^+^ Kupffer cells in our current and previous studies [10] as well as to bone marrow derived hepatic macrophages which Klein et al. proposed [11]. This Kupffer cell population with TNF producing capacity may thus play pivotal role in various inflammatory responses in the liver.

A previous study reported that NKT cells may be main TNF producers after α-GalCer injection [23]. However, using a similar intracellular TNF staining method, we demonstrated that CD11b^+^ Kupffer cells stimulated with α-GalCer are main TNF producers, whereas NKT cells may produce a low amount of TNF. The precise reason of the descrepancy is unknown at present. However, since the serum TNF level peaks 1 h after α-GalCer administration, we harvested liver MNCs from mice 30 min after α-GalCer injection, whereas they took out liver MNCs from mice 1.5 h after injection. Nevertheless, it was also difficult for us to clearly show intracellular TNF after α-GalCer injection when using CD mice. In addition, a flowcytometric analysis of macrophages/Kupffer cells is difficult as compared to that of lymphocytes because non-specific staining has to always be excluded by isotype controls and the adjustment of voltage and compensation of flow cytometer is sometimes needed among the different types of staining.

Immunology has recently been recognized to play important roles not only in the host defense against microbes, but also in the induction of many diseases other than classical immunology-related autoimmune diseases and allergic disorders. For example, immunological mechanisms are now known to be involved in atherosclerosis and associated cardiovascular diseases [24], [25]. Macrophages phagocytose oxidized LDL, become foam cells, and move into the intima of arterial walls [25], [26]. These macrophages/foam cells may be activated by bacteria or their components to produce proinflammatory cytokines, and can then activate T cells and NKT cells [27]–[29]. These inflammatory reactions may induce tissue damage and smooth muscle cell accumulation in the arterial intima, thus resulting in atherosclerosis.

NASH is now recognized as one of the major causes of cirrhosis and subsequent hepatocellular carcinoma [30]. A “two hits” theory was proposed as the mechanism underlying the development of NASH [31]. Hypercholesterolemia in the liver may be a first hit (priming), which increases the sensitivity of the hepatocytes to a second hit, and the second hits for NASH [31] may include TNF or ROS (or their combination) produced by Kupffer cells and/or hepatocytes, which may be induced by bacterial infections and other unknown mechanisms.

Although Kupffer cells have been suggested to be involved in the onset of NASH [32], the role of innate immune lymphocytes in the liver in relation to Kupffer cells has been unclear. The results of CpG-induced hepatitis in this study suggest that every bacterial infection can be a second hit. Even relatively minor pathologic events—such as increased bacterial antigens from the intestine, covert bacterial infections, or other events (as a second hit) in humans with hypercholesterolemia may induce NASH, in which either apoptosis, necrosis, or their combination may be observed. Our present results showed that the liver cholesterol levels, but not the liver triglyceride levels, serum cholesterol levels or body weight, may profoundly be involved in hepatocyte injury, which may explain why there are people with simple liver steatosis without inflammatory response and patients with NASH with mild to severe hepatocyte injury. Consistent with the current study, our recent study demonstrated that hepatic injury during the Shwartzman reaction (IL-12 and LPS injection 16 h apart) was aggravated in HFCD mice compared to CD mice, and that NK cells/NKT cells killed vascular endothelial cells (although Fas/FasL independent manner) [13]. We have proposed that human liver CD56^+^ T cells but not Vα24^+^ T cells are functional counterpart of mouse liver NKT (Vα14^+^) cells [2], [33], [34], and CD56^+^ T cells with a potent FasL producing capacity [33], [35] may also be involved in NASH.

Notably, injection of α-GalCer into partially hepatectomized mice accelerates hepatocyte proliferation and liver regeneration in a TNF/FasL-dependent manner [36]. TNF/Fas may bind to the TNF receptor, and subsequently to the TNF receptor-associated death domain (TRAD). TNF/Fas may then activate the Fas-associated death domain (FAAD) in old or damaged hepatocytes, leading to apoptosis. TNF may also activate the TNF receptor-associated factor (TRAF) and receptor interacting protein (RIP), which are associated with NF-κB activation in young and regenerating hepatocytes, leading to their activation and proliferation. The TNF/FasL (NKT cells)/Fas pathway may thus be a crucial physiological system which primarily maintains the homeostasis of hepatocytes.

Finally, it should be noted that, since hepatocyte injuries in humans may also be caused by TNF/FasL-dependent apoptosis, it follows that anti-TNF Abs and anti-FasL Abs should be considered for therapeutic strategies for some NASH patients. In fact, a NASH patient was reported who experienced a rapid normalization of liver injury during the treatment of associated rheumatoid arthritis with a humanized anti-TNF Ab [37].

## Supporting Information

Figure S1 **Body weights, liver triglyceride levels, serum total cholesterol levels and liver cholesterol levels in mice.** CD mice, HFD mice, CD mice and HFCD mice were fed each diet for four weeks and were examined. The data shown are the means ± SE from six to ten mice in each group. a, b, c; *P<0.05 vs CD and HCD, d; *P<0.05 vs CD and HFD, ** *P*<0.01 vs. CD and HFD.
(EPS)

Figure S2 **CD1d expression by CD68 Kupffer cells.** Liver MNCs were obtained from CD, HFD, HCD and HFCD mice, and Kupffer cells gated by F4/80 and CD68 were analyzed for their expression of CD1d. The data shown are representatives of three mice with similar results.
(EPS)

Figure S3 (a, b, c) The effect of the depletion of NK/NKT cells or NK cells alone on *in vitro* TNF, IFN-γ and IL-4 production in CD mice after α-GalCer stimulation. Depletion of NK cells and NKT cells or NK cells alone was performed as described in materials and methods. The data are the means± SE from three mice in each group. (d, e, f) Comparion of cytokine production among total liver MNCs, F4/80(CD11b)^+^ cells and F4/80(CD11b)^−^ cells from HFCD mice. Liver MNCs were harvested from eight to ten HFCD mice without collagenase digestion and F4/80^+^ cells (mostly CD11b^+^) were purified by a MACS sorting system. A total of 5×10^5^ F4/80^+^ population/200 µl were stimulated with either α-GalCer or CpG in a 96 well plate for 6 h for TNF and for 24 h either for IFN-γ or IL-4. The supernatants were examined for TNF, IFN-γ and IL-4. The data shown are the means ± SE from three independent experiments. *P<0.05.
(EPS)

Figure S4 **Intracellular TNF expression by liver Vβ8^+^T cells with intermediate TCR (NKT cells).** Liver MNCs from CD mice, HFCD mice and HFCD mice (3 months) were obtained 30 min after α-GalCer injection without collagenase treatment and liver Vβ8^+^ cells with intermediate TCR were examined for their intracellular TNF staining. Data are the representative findings from three to five mice with similar results.
(EPS)

Figure S5 **Reversion of the the proportion of CD11b^+^ subset in the total F4/80 Kupffer cell population in the livers of HFCD/CD mice.** The mice received HFCD for four weeks, and then the HFCD diet was changed to a CD diet (HFCD/CD) for additional four weeks, and F4/80-gated liver MNCs were analyzed by flow cytometry. The numbers were means±SE from four mice in each group.
(EPS)
